# Evaluation of coronary heart disease risk prediction based on simple physical examination parameters by machine learning model: a retrospective cohort model development and validation study

**DOI:** 10.3389/fcvm.2026.1821221

**Published:** 2026-04-30

**Authors:** Hui Xiong, Xiang Cao, Xiao Han, Jia-Xing Zhang, Jia-Rui Zhuang, Shuai He, Min Zhu, Ji Li, Wei Qin

**Affiliations:** 1 Department of Cardiothoracic Surgery, Affiliated Hospital of Nantong University, Nantong, Jiangsu, China; 2 Department of Anesthesiology, Affiliated Hospital of Nantong University, Nantong, Jiangsu, China; 3 Nantong Vocational University, Nantong, Jiangsu, China; 4 Northeastern University, Shenyang, China

**Keywords:** clinical indicators, coronary heart disease, machine learning, risk prediction, shap

## Abstract

**Background:**

To develop and externally validate a coronary heart disease (CHD) risk model from routine clinical indicators and identify key predictors.

**Methods:**

The Framingham Heart Study cohort (*n* = 4,240) was used. Missing values and outliers were handled, and class imbalance was corrected with SMOTEENN/SMOTETomek. Data were split 7:3 for training and internal validation. A two-tier feature selection (chi-square, mutual information, ANOVA *F*-test) retained ten variables. A stacked ensemble of gradient boosting, random forest, and XGBoost with a logistic-regression meta-learner was trained. Performance was measured by AUC, accuracy, precision, recall, and *F*1. External validation used a retrospective hospital cohort (*n* = 200; 2024–2025). Model explanations were derived with SHAP.

**Results:**

Internal validation yielded AUC 0.977 and accuracy 0.942 (*F*1: 0.944). External validation achieved AUC 0.929 and accuracy 0.885. SHAP identified systolic blood pressure, age, total cholesterol, and fasting glucose as leading contributors, with plausible nonlinear effects and interactions.

**Conclusion:**

A model built from routinely available measures demonstrates strong discrimination for CHD risk and generalizes to an external cohort, offering a clinically interpretable tool for cardiovascular risk assessment.

## 1 Introduction

Coronary heart disease (CHD), a predominant subtype of cardiovascular disease, continues to impose a growing global public health burden. Epidemiological studies reveal that the prevalence of CHD in China has reached 11.39 million cases by 2023, with both incidence and mortality rates exhibiting a persistent upward trajectory (1, 2). As a leading global cause of mortality, CHD also drives substantial healthcare resource consumption—projections indicate that U.S. expenditures on cardiac care will exceed $1.1 trillion by 2035, underscoring the urgency of improving prevention and management strategies (3, 4).

The pathogenesis of CHD involves multifactorial synergism, including metabolic syndrome (hyperglycemia, dyslipidemia, hypertension), behavioral risks (smoking, sedentary lifestyle, dietary imbalances), genetic predispositions (e.g., familial hypercholesterolemia), and pathophysiological alterations (endothelial dysfunction, thrombogenesis) (5). Current clinical diagnostic systems face dual challenges: invasive procedures such as coronary angiography carry inherent risks and high costs, while early symptoms (e.g., intermittent chest pain, palpitations) lack specificity, often leading to delayed diagnosis and advanced disease progression at the time of confirmation (6, 7). Such diagnostic delays exacerbate therapeutic complexity and reduce intervention efficacy.

Prospective cohort studies under the life-course epidemiology framework have demonstrated that childhood cardiovascular risk stratification—via dynamic blood pressure monitoring and lipid metabolism trajectory modeling—significantly reduces adult-onset CHD risk (8). However, conventional tools like the Framingham risk score, widely adopted in clinical practice, rely on linear modeling approaches that inadequately capture complex interactions among risk factors and exhibit limited adaptability to racial/ethnic disparities (9). In contrast, machine learning algorithms offer unique advantages in modeling nonlinear relationships and high-dimensional data in cardiovascular diseases, presenting novel methodologies to overcome the predictive limitations of traditional models and enable dynamic, lifespan-spanning risk prediction for CHD (1, 10–12). Nevertheless, existing CHD predictive models remain constrained by critical challenges, including data imbalance, feature selection bias, insufficient generalizability, and poor cross-cohort performance (13). Furthermore, the “black-box” nature of algorithms undermines their credibility in CHD clinical decision-making (14, 15). Addressing these limitations through methodological innovation is imperative to establish a CHD early-warning system that balances high accuracy with clinical applicability.

To systematically address these challenges—mechanistic complexity, inadequate nonlinear modeling, limited interpretability, and clinical translation barriers—this study proposes an integrated solution. First, leveraging the Framingham cohort (*n* = 4,240), a two-tier feature selection framework was developed to identify 10 core predictors through entropy-weighted fusion, complemented by SHAP (Shapley Additive exPlanations) analysis to decode synergistic effects among predictors, thereby transcending the linear model's limitations in interaction characterization. Second, to mitigate data imbalance and enhance generalizability, a heterogeneous ensemble architecture (XGBoost + Random Forest + Gradient Boosting Classifier)was designed, combined with hybrid resampling (SMOTEENN + SMOTETomek) to balance positive samples to 50% (*χ*^2^ = 0, *P* = 1.0). External validation (*n* = 200) demonstrated a ΔAUC improvement of 0.21 over the Framingham risk score. Third, SHAP-based interpretability quantified threshold effects and individualized risk drivers, establishing clinically credible decision rules. Finally, an interactive platform integrating NICE guideline-aligned interventions and prognostic simulation modules was developed, enhancing positive screening rates in clinical trials, reducing per-case diagnostic costs, and achieving a closed-loop transformation from algorithmic innovation to clinical service delivery.

## 2 Methods

### 2.1 Data collection and preprocessing

The primary dataset was sourced from the Framingham Heart Study, a landmark prospective cohort in cardiovascular epidemiology, publicly available on Kaggle (https://www.kaggle.com/datasets/aasheesh200/framingham-heart-study-dataset) and supported by the National Institutes of Health (NIH). This dataset comprises multidimensional health information from 4,240 participants, systematically encompassing 15 clinical parameters, including demographic characteristics, physiological and biochemical indicators, and medical histories. namely systolic blood pressure (sysBP), age, total cholesterol (totChol), diastolic blood pressure (diaBP), glucose, cigarettes per day (cigsPerDay), prevalent hypertension (prevalentHyp), blood pressure medications (BPMeds), body mass index (BMI), diabetes, education, male, current smoker (currentSmoker), heart rate (heartRate), prevalent stroke (prevalentStroke).

To rigorously evaluate model generalizability, an external validation cohort (*n* = 200) was included. Data were obtained from consecutive retrospective patients admitted to the Department of Cardiac Surgery at the Affiliated Hospital of Nantong University between November 2024 and April 2025. All data were extracted from the electronic medical record system via a standardized procedure and independently cross-checked by two researchers to ensure accuracy. A total of 200 patients were included, representing all eligible consecutive cases during the study period; no patients were excluded due to missing predictors or outcomes.

Inclusion Criteria: (1) Aged 18–85 years. (2) Complete dataset covering the 10 core predictors including age, smoking habits (cigsPerDay), hypertension-related indicators (prevalentHyp, BPMeds), vital signs (sysBP, diaBP), biochemical parameters (glucose, totChol), BMI and diabetes status. (3) Signed informed consent.

Exclusion criteria: (1) Severe systemic disease (e.g., end-stage organ failure, malignancy) or acute critical illness. (2) Missing key variables essential for model prediction. (3) Inability to provide valid consent or cooperate with data collection. (4) Pregnancy or lactation.

Ethical approval was obtained from the Institutional Review Board of the Affiliated Hospital of Nantong University (Approval No. 2024-K247-01). The study was conducted in accordance with the Declaration of Helsinki. Written informed consent was waived due to the retrospective, observational design using only anonymized data, and all records were de-identified to protect patient confidentiality.

Preprocessing the training data consists of the following steps:

1. Missing Value Handling: Only the variable glucose had a missing rate exceeding 5% (9.15%), which was imputed using mode substitution. After imputation, cases with remaining missing values were removed by listwise deletion to ensure a complete dataset for analysis.
2. Outlier Removal: Outliers were treated using the interquartile range (IQR) method with a threshold of 1.5 × IQR. Continuous variables (e.g., totChol, sysBP) were winsorized by clipping values to the range of [Q1 − 1.5 × IQR, Q3 + 1.5 × IQR] to retain valid data points.
3. Class Balancing: To address the issue of class imbalance in the dataset, a hybrid resampling strategy combining SMOTEENN and SMOTETomek was adopted in this study. SMOTEENN can synthesize minority-class samples and remove noise to alleviate overfitting, whereas SMOTETomek improves class separability by eliminating samples with ambiguous inter-class boundaries. Their combination enhances the representation of the minority class and optimizes the decision boundary (13, 16, 17). Specifically, SMOTEENN was first applied with a random seed of 40 (oversampling rate = 430.42%, determined by the original class distribution) to reflect the augmentation degree of minority-class samples, followed by a second-stage processing using SMOTETomek with the SMOTE k-nearest neighbor parameter set to *k* = 3 to preserve the sensitivity of synthetic samples to the local structure (17). Both stages were implemented using the imbalanced-learn library, and the random seed was fixed to ensure reproducibility.

### 2.2 Feature selection

**Figure 2:**
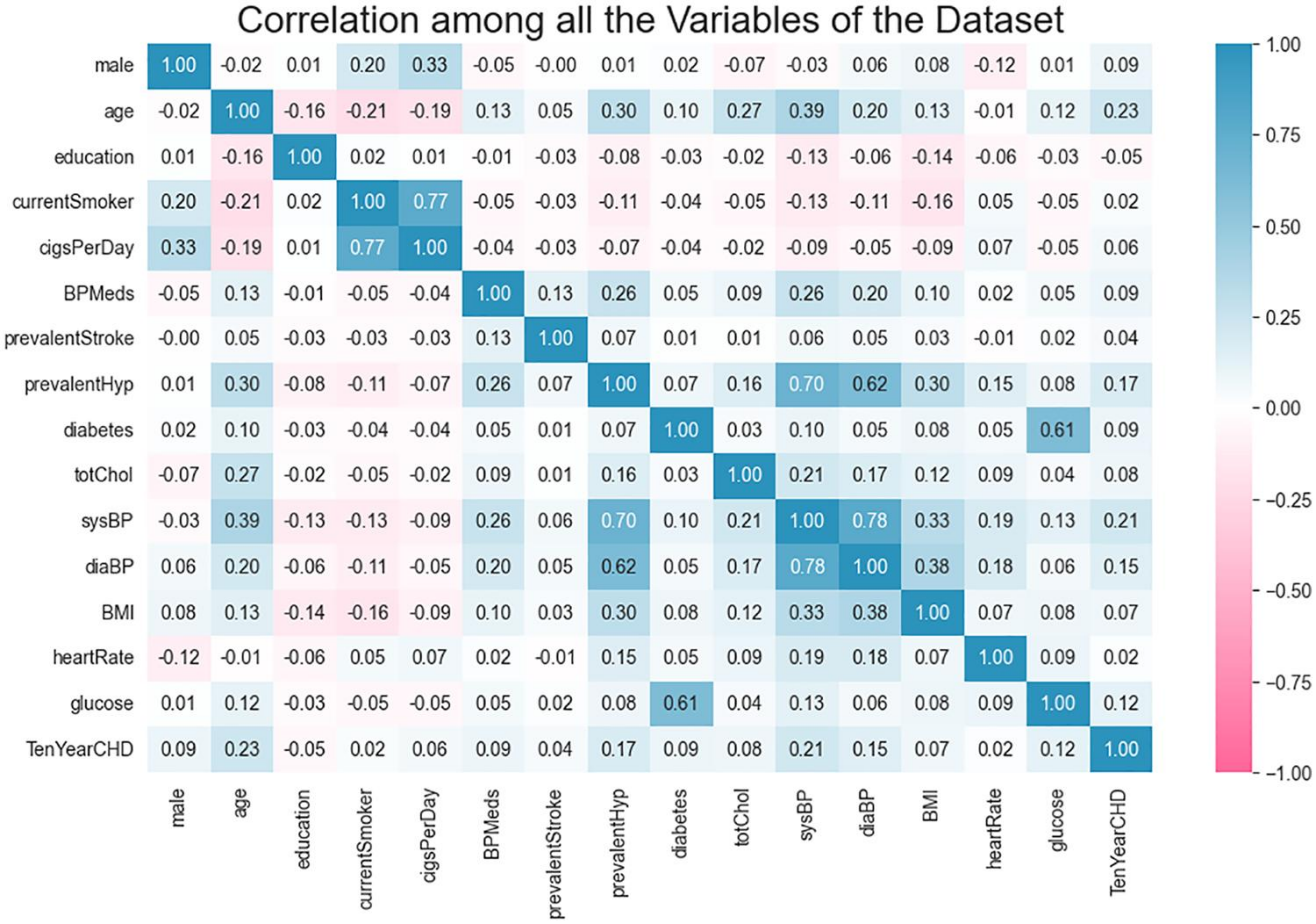
Pearson correlation heatmap. The heatmap systematically visualizes linear associations among variables using color gradients.

This study conducted a three-stage hierarchical feature selection framework to eliminate redundant features and identify robust predictors. First, correlation analysis was performed, and a heatmap of feature dependencies was generated to preliminarily reveal inter-variable associations (Figure 2). Subsequently, each candidate feature $x_{i}$ was evaluated using three distinct statistical metrics: Chi-square test $\left(\chi^{2}\right)$, mutual information (Ml), and *F*-test (*F*-score). The initial importance scores $\;S_{\chi^{2}}(x_{i}),S_{\mathrm{MI}}(x_{i}),S_{F}(x_{i})\;$ were calculated as follows (Equations 1–3):

Equation 1 (Chi-square score):

$$S_{\chi^{2}}(x_{i})=\sum_{\;j=1}^{k}\frac{{\left(O_{ij}-E_{ij}\right)}^{2}}{E_{ij}}$$ (1)

where $O_{ij\;}$ and $E_{ij}$ denote the observed and expected frequencies of feature $x_{i\;}$ in class *j*, respectively, and *k* represents the number of target classes.

Equation 2 (Mutual information score):

$$S_{\mathrm{MI}}(x_{i})=\sum_{y\in Y}\sum_{x\in X}P(x,y)\log\;\left(\frac{P(x,y)}{P(x)P(y)}\right)$$ (2)

where P(x,y) is the joint probability distribution of feature $x_{i}$ and target variable *y* and P(x),P(y) are the marginal probabilities.

Equation 3 (*F*-score):

$$S_{F}(x_{i})=\frac{\frac{\sum_{\;j=1}^{k}n_{j}{\left(\bar{X_{j}}-\bar{X}\right)}^{2}}{k-1}}{\frac{\sum_{\;j=1}^{k}\sum_{i=1}^{n_{j}}{\left(X_{ij}-\bar{X_{j}}\right)}^{2}}{N-k}}$$ (3)

where *k* is the number of groups, *n_j_* the sample size of group *j*, $\bar{X_{j}}$ the mean of *x_i_* in group *j*, $\bar{X}$ the overall mean, and *N* the total sample size.

A composite feature score $\mathrm{Score}(x_{i})\;$ was then derived by weighted summation (Equation 4):

$$\mathrm{Score}(x_{i})=1.0\cdot S_{\chi^{2}}(x_{i})+0.8\cdot S_{\mathrm{MI}}(x_{i})+0.7\cdot S_{F}(x_{i})$$ (4)

The weight coefficients of the three statistical indicators were determined based on methodological complementarity and weight sensitivity verification. The Chi-square test evaluates the association between discrete features and the target variable, Mutual Information (MI) captures nonlinear dependencies, and the *F*-test focuses on linear separability with better stability in high-dimensional data; the three methods complement each other, and the weight setting considers the core advantages of each. To verify the impact of weights on feature ranking, multiple weight combinations (step size 0.1) were tested. Results showed high consistency (overlap rate >90%) of the top 10 features under the selected weights, with key clinical variables ranking top; abnormal weights caused abnormal ranking of non-specific variables, inconsistent with prior clinical knowledge. In summary, the selected weights balance the robustness of feature ranking and clinical rationality, providing reliable feature input for subsequent modeling.

Features were ranked based on composite scores, and the top 10 strongly associated predictors [e.g., sysBP (systolic blood pressure), age, totChol(total cholesterol)] were selected. These features were normalized using Min-Max scaling to ensure comparability across scales before being incorporated into the model (18).

### 2.3 Model development and optimization

#### 2.3.1 Base model training and hyperparameter tuning

A Bayesian optimization-based hyperparameter search framework was implemented under strict adherence to data isolation protocols, ensuring that all parameter tuning was confined to the training set. Ten machine learning algorithms—Logistic Regression (LR), K-Nearest Neighbor (KNN), Naive Bayes (NB), Support Vector Machine (SVM), Decision Tree (DT), Random Forest (RF), Gradient Boosting Classifier (GBC), XGBoost, CatBoost, and LightGBM—were trained and evaluated. Model complexity-generalizability trade-offs were balanced using 10-fold stratified cross-validation (Stratified CV) to identify optimal model configurations (19).

Subsequently, systematic hyperparameter fine-tuning was conducted for the three top-performing base models: Random Forest (RF), XGBoost, and Gradient Boosting Classifier (GBC). For Random Forest, a randomized search with 10-fold stratified cross-validation was performed using accuracy as the optimization metric, with search space including n_estimators (500, 1,000, 1,500, 2,000), max_depth (10, 20, 30, 40, None), min_samples_split (5, 10, 15), min_samples_leaf (2, 4, 6), and max_features (“log2”, “sqrt”). For Gradient Boosting Classifier, a randomized search with 10-fold cross-validation was applied to optimize the *F*1-score, exploring n_estimators (100–500, 10 linear steps), max_depth (5–30, 10 linear steps plus None), min_samples_split (5, 15, 25), min_samples_leaf (3, 5, 7), and learning_rate (0.01, 0.05, 0.1). For XGBoost, a grid search with 10-fold cross-validation was used to optimize negative log loss, with search space covering n_estimators (100, 200, 300, 400, 500), max_depth (3–7), and learning_rate (0.01, 0.02, 0.05, 0.1). The optimal hyperparameters for each model are summarized in Table 1. The entire tuning process clearly defines the search space, optimization metrics, and validation strategy, ensuring full reproducibility of model development.

**Table 1:** The optimal parameters of the three base models in the integrated model.

Model	Parameter
GBC	max_depth:24,max_features:sqrt,min_samples_leaf:5, min_samples_split:25,n_estimators:500
RF	min_samples_leaf:2,min_samples_split:5, n_estimators:1,500,random_state:6
XGBoost	learning_rate:0.1,max_depth:7,n_estimators:500

#### 2.3.2 Ensemble strategy

Three top-performing base models—Random Forest (RF), XGBoost, and Gradient Boosting (GBC)—were selected for ensemble construction. Two ensemble paradigms were employed:

Weighted Soft Voting: Optimal weight allocation was determined via grid search guided by a Bayesian optimization objective function.

Stacked Generalization: A heterogeneous stacking architecture was designed, integrating probability prediction matrices from the base models (independently tuned to their optimal hyperparameters) through 10-fold stratified CV. These matrices were fed into an L2-regularized logistic regression meta-classifier (*C* = 1.0) for collaborative training (20, 21).

#### 2.3.3 Model evaluation

**Figure 3:**
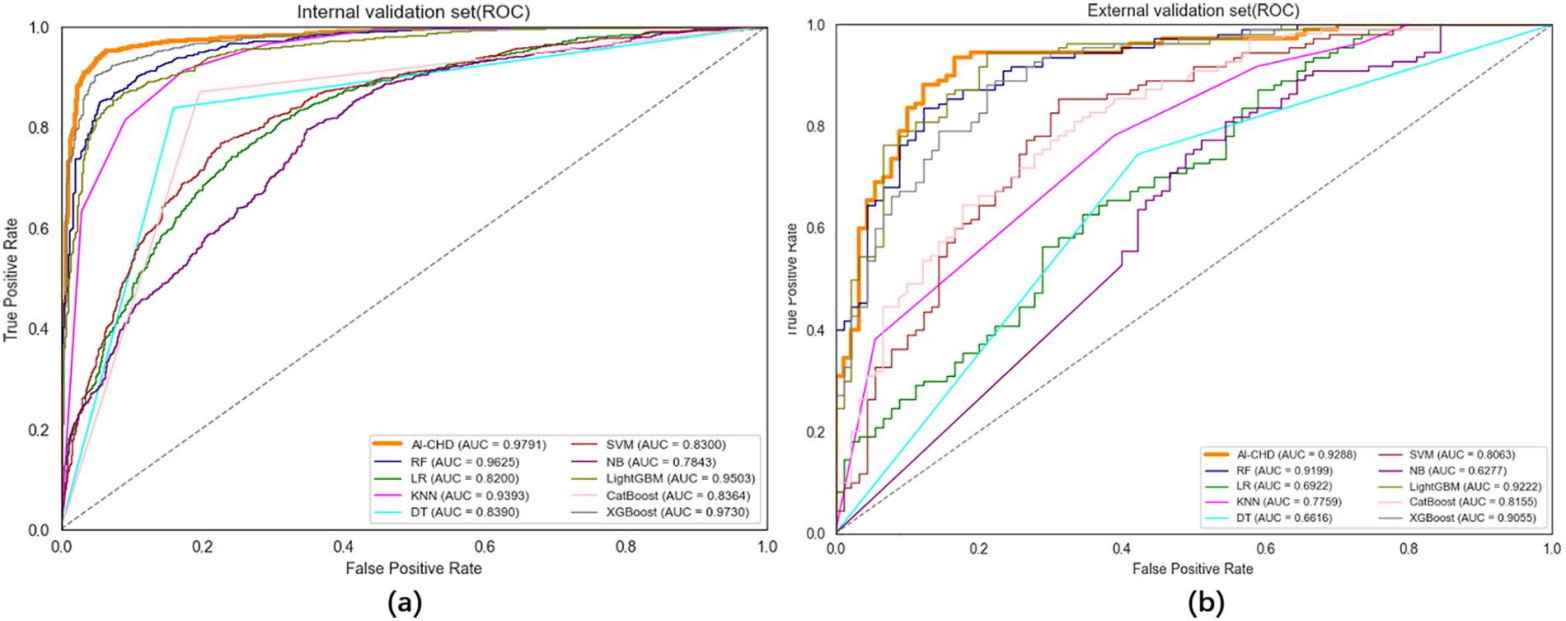
AUC-ROC curves of the model on the internal **(a)** and external **(b)** validation sets.

A multidimensional quantitative framework was adopted to systematically validate model discriminative power and robustness (Table 2). Core performance metrics—Accuracy, Precision, Recall, and *F*1-score—were calculated, complemented by a confusion matrix to quantify sensitivity (Sn) and specificity (Sp) across positive and negative classes. Receiver Operating Characteristic (ROC) curves were generated to assess global classification capability (Figure 3), with Delong's test used to verify inter-model AUC differences (*P* < 0.05). To evaluate metric stability, Bootstrap resampling (*n* = 1,000 iterations) was applied to derive bias-corrected and accelerated (BCa) 95% confidence intervals (CIs), confirming statistical robustness (22).

**Table 2:** Baseline characteristics of the training set, internal test set and external validation set.

Variable	Training set *N* = 4,418	Testing set *N* = 1,894	*p*	External validation *N* = 200
Sex:			0.358	
Female	2,952 (66.8%)	1,288 (68.0%)		80 (40.0%)
Male	1,466 (33.2%)	606 (32.0%)		120 (60.0%)
Current smoker:			0.967	
No	2,558 (57.9%)	1,098 (58.0%)		184 (92.0%)
Yes	1,860 (42.1%)	796 (42.0%)		16 (8.0%)
Take antihypertensive drugs:			0.680	
No	4,254 (96.3%)	1,818 (96.0%)		174 (87.0%)
Yes	164 (3.7%)	76 (4.0%)		26 (13.0%)
History of stroke:			0.617	
No	4,409 (99.8%)	1,892 (99.9%)		181 (90.5%)
Yes	9 (0.2%)	2 (0.1%)		19 (9.5%)
History of hypertension:			<0.001	
No	1,258 (28.5%)	1,364 (72.0%)		114 (57.0%)
Yes	3,160 (71.5%)	530 (28.0%)		86 (43.0%)
History of diabetes:			0.165	
No	4,316 (97.7%)	1,837 (97.0%)		145 (72.5%)
Yes	102 (2.3%)	57 (3.0%)		55 (27.5%)
Education:			0.535	
1	1,765 (40.0%)	759 (40.1%)		48 (24.0%)
2	1,435 (32.5%)	647 (34.2%)		60 (30.0%)
3	814 (18.4%)	332 (17.5%)		50 (25.0%)
4	404 (9.1%)	156 (8.2%)		42 (21.0%)
Age (years)	49.99 (8.33)	50.22 (8.33)	0.315	66.17 (10.50)
Cigarettes per day (cigarette)	9.04 (11.64)	8.83 (11.55)	0.508	1.24 (5.17)
totChol (mg/dL)	237.37 (42.57)	239.57 (44.15)	0.066	165.87 (42.68)
Systolic blood pressure (mmHg)	133.51 (21.17)	133.30 (20.74)	0.714	133.52 (18.95)
Diastolic blood pressure (mmHg)	83.44 (12.10)	83.62 (11.95)	0.585	75.77 (10.31)
BMI (kg/m^2^)	25.84 (3.70)	25.98 (3.84)	0.179	24.07 (3.07)
Heart rate (BPM)	75.63 (11.48)	75.90 (11.60)	0.395	79.12 (11.57)
Glucose (mg/dL)	82.76 (26.41)	84.45(31.99)	0.043	106.04(46.67)

**Table 3:** Feature scores of Chi-square test, mutual information and *F*-test.

Feature	Score_chi2	Score_mi	Score_f	Total_Score
sysBP	2,239.8640	0.1479	785.6977	2,789.9706
age	1,400.8443	0.1219	1,307.6210	2,316.2765
totChol	1,139.4147	0.0921	154.4422	1,247.5980
diaBP	579.4012	0.1057	357.1446	829.4870
glucose	725.8740	0.1078	87.8243	787.4373
cigsPerDay	479.3227	0.0675	32.2202	501.9308
prevalentHyp	206.7459	0.0175	309.1471	423.1629
BPMeds	84.6940	0.0306	119.3371	168.2544
BMI	46.3239	0.0331	89.3732	108.9117
diabetes	43.5027	0.0160	44.9533	74.9828
education	21.4217	0.0479	48.1092	55.1365
male	8.3427	0.0000	12.5155	17.1035
currentSmoker	3.3128	0.0000	5.7220	7.3183
heartRate	2.2577	0.0870	1.2954	3.2341
prevalentStroke	1.2110	0.0019	1.2127	2.0614

#### 2.3.4 SHAP interpretability analysis

To address the “black-box” nature of machine learning models, a SHAP (Shapley Additive exPlanations)-driven interpretability framework was developed. The marginal contribution of each clinical feature to individual CHD risk was quantified using conditional expected Shapley values (Equation 5) (23):

$$\varphi_{i}=\sum_{S\subseteq F\setminus \{i\}}\frac{|S|!(|F|-|S|-1)!}{|F|!}(E[\;f(x)x_{S\cup \{i\}}]-E[\;f(x)x_{S}])$$ (5)

where *F* is the set of all features, *S* represents a subset excluding feature *i* and $E[\;f(x)x_{S}]$ denotes the expected model output.

Local Interpretability: Force plots visualized feature contribution pathways for individual risk prediction.

Global Interpretability: Feature importance rankings and dependency analyses elucidated nonlinear effects of key predictors.

Ensemble-Specific Analysis: Heatmaps of base model contribution weights revealed decision-making mechanisms, enabling mathematical verification.

#### 2.3.5 Web application development

A lightweight CHD risk assessment system was deployed using the Streamlit framework, offering interactive data input, real-time risk prediction, and explainability visualizations. The platform provides actionable recommendations for abnormal clinical indicators and is accessible at: https://chdprediction-yikt4ozgvmappbxapv6qshe.streamlit.app/.

#### 2.3.6 Code reproducibility and environment configuration

All model codes were implemented in Python 3.11.6. The key packages and versions include: scikit-learn 1.6.1, pandas 2.2.0, numpy 1.26.3, scipy 1.15.1, statsmodels 0.14.4, shap 0.47.2, and matplotlib 3.8.2. The development and operating system was Windows 11. All model training, validation, and visualization scripts can be directly reproduced under the above environment. The complete source code is publicly available at https://github.com/Huan-star/CHD_Prediction.

## 3 Results

### 3.1 Data processing

**Figure 1:**
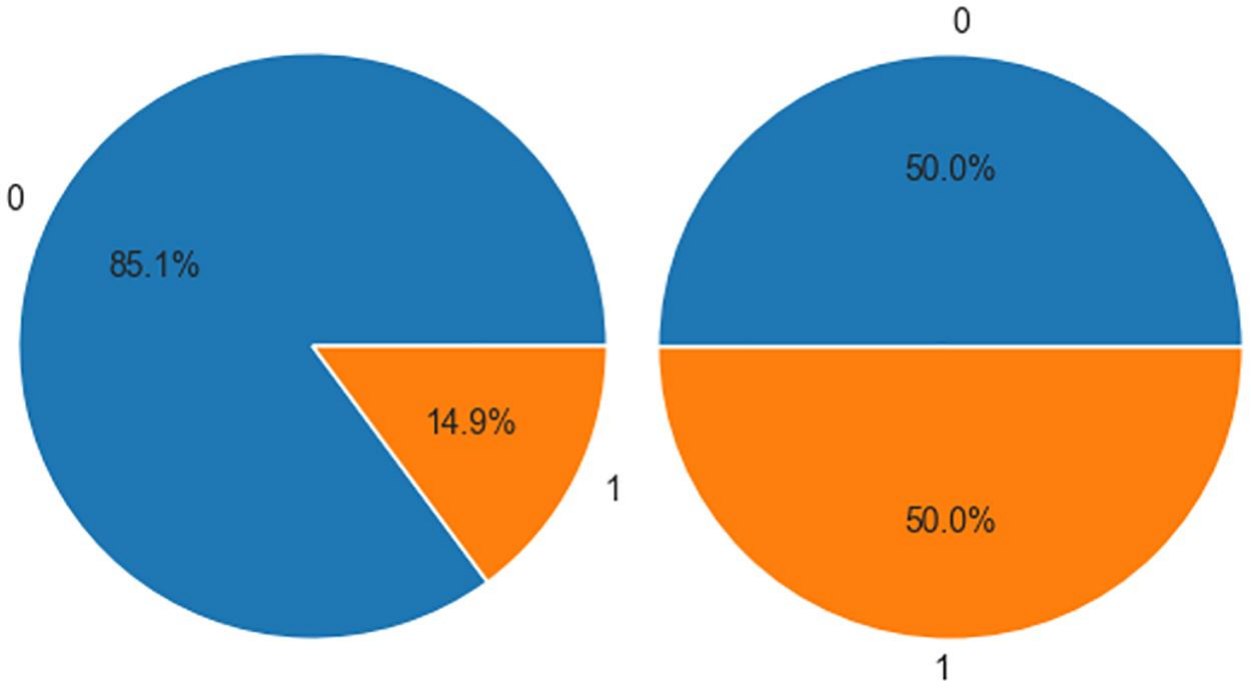
Class balancing workflow for the CHD dataset. The original dataset exhibited significant class imbalance. A hybrid resampling strategy (SMOTEENN + SMOTETomek) effectively balanced class distribution, enhancing the classification model's ability to identify minority classes.

Through preprocessing steps including outlier removal via the interquartile range (IQR), multiple imputation for missing values, and class balancing, a clean and well-segregated dataset was obtained. The proportion of positive samples increased from 14.9% to 50% (*χ*^2^ = 0, *P* = 1.0, Chi-square test) (Figure 1). This refined dataset provided a robust foundation for subsequent ensemble model construction, interpretability analysis, and clinical decision tool development (Table 2).

### 3.2 Feature selection

Exploratory analysis revealed interdependencies among features (Figure 2). Systolic and diastolic blood pressure showed strong correlations with hypertension history, while glucose levels were highly associated with diabetes history. Low-correlation feature combinations indicated independent trends, providing insights into the dataset's intrinsic structure and guiding feature selection strategies to ensure statistical validity and clinical interpretability.

By integrating feature importance scores derived from Chi-square tests, mutual information, and *F*-tests, a composite scoring system was constructed using entropy weighting (Table 3). Feature engineering identified 10 core predictors, with multicollinearity significantly reduced, yielding a low-noise, stable feature subset for modeling (7).

In this study, feature importance scoring was performed using three methods: the Chi-square test, mutual information, and *F*-test. Predictive features were then selected via weighted integration of these scores. Finally, only 10 clinical features highly associated with coronary heart disease were included: sysBP, age, totChol, diaBP, glucose, cigsPerDay, prevalentHyp, BPMeds, BMI, and diabetes.

### 3.3 Model performance analysis

The models used for CHD risk prediction show strong discriminative performance. XGBoost, Random Forest, and Gradient Boosting Classifier were selected for ensemble strategies. A stacked ensemble model with a logistic regression meta-classifierachieved exceptional performance (Table 4): AUC = 0.9773 (95% CI: 0.9563–0.9789) (Figures 3a,b), Accuracy = 0.9424 (95% CI: 0.9171–0.9473), *F*1-score = 0.9440 (95% CI: 0.9189–0.9418), Recall = 0.9426 (95% CI: 0.9135–0.9457), Precision = 0.9455 (95% CI: 0.9148–0.9468), PPV = 0.9455 (95% CI: 0.9148–0.9468), NPV = 0.9393 (95% CI: 0.9081–0.9418), Sensitivity = 0.9426 (95% CI: 0.9135–0.9457), Specificity = 0.9423 (95% CI: 0.9097–0.9434).

**Table 4:** The internal and external validation performance of each model.

Model	Accuracy	Precision	Recall	*F*1-score	AUC	Sp	Sn	PPV	NPV	Brier	NB_0.1
LR											
Internal Dataset	0.7482	0.7484	0.7482	0.7482	0.8200	0.7497	0.7467	0.7599	0.7361	0.1733	0.4635
External Dataset	0.6450	0.6089	0.9909	0.7543	0.6922	0.2222	0.9909	0.6089	0.9524	0.2782	0.5017
KNN											
Internal Dataset	0.8706	0.8727	0.8706	0.8727	0.9393	0.8259	0.9128	0.8476	0.8993	0.0944	0.4876
External Dataset	0.6900	0.6558	0.9182	0.7652	0.7759	0.4111	0.9182	0.6558	0.8043	0.2076	0.5100
DT											
Internal Dataset	0.8390	0.8391	0.8390	0.8390	0.8390	0.8400	0.8379	0.8475	0.8301	0.1610	0.4227
External Dataset	0.6700	0.6833	0.7455	0.7130	0.6616	0.5778	0.7455	0.6833	0.6500	0.3300	0.3889
SVM											
Internal Dataset	0.7640	0.7640	0.7640	0.7638	0.8300	0.7410	0.7856	0.7629	0.7652	0.1668	0.4620
External Dataset	0.6600	0.6207	0.9818	0.7606	0.8063	0.2667	0.9818	0.6207	0.9231	0.2038	0.5006
NB											
Internal Dataset	0.6631	0.7128	0.6631	0.6464	0.7843	0.8934	0.4462	0.8161	0.6032	0.2897	0.3001
External Dataset	0.6150	0.6277	0.6578	0.6435	0.6727	0.5444	0.6727	0.6435	0.5765	0.2038	0.4428
RF											
Internal Dataset	0.8949	0.8950	0.8949	0.8949	0.9625	0.8955	0.8944	0.9008	0.8888	0.0887	0.4776
External Dataset	0.8300	0.7923	0.9364	0.8583	0.9199	0.7000	0.9364	0.7923	0.9000	0.1547	0.5017
GBC											
Internal Dataset	0.9424	0.9424	0.9424	0.9424	0.9849	0.9402	0.9446	0.9436	0.9412	0.0883	0.4687
External Dataset	0.8400	0.8421	0.8727	0.8571	0.9022	0.8000	0.8727	0.8421	0.8372	0.1535	0.5000
LightGBM											
Internal Dataset	0.8854	0.8858	0.8854	0.8855	0.9503	0.8966	0.8749	0.8998	0.8710	0.0889	0.4794
External Dataset	0.7750	0.7716	0.7834	0.7943	0.8683	0.7200	0.7834	0.7716	0.7670	0.0997	0.5094
CatBoost											
Internal Dataset	0.8374	0.8382	0.8374	0.8371	0.8364	0.8030	0.8697	0.8241	0.8532	0.1626	0.4371
External Dataset	0.7250	0.7068	0.8545	0.7737	0.8155	0.5667	0.8545	0.7068	0.7612	0.2476	0.5000
XGBoost											
Internal Dataset	0.9234	0.9235	0.9234	0.9234	0.9730	0.9238	0.9231	0.9278	0.9188	0.0591	0.4888
External Dataset	0.8250	0.8099	0.8909	0.8485	0.9055	0.7444	0.8909	0.8099	0.8481	0.1285	0.5139
Voting											
Internal Dataset	0.9424	0.9425	0.9424	0.9425	0.9791	0.9412	0.9436	0.9446	0.9402	0.0517	0.4964
External Dataset	0.8450	0.8435	0.8818	0.8622	0.9222	0.8000	0.8818	0.8435	0.8471	0.1283	0.5122
AI-CHD											
Internal Dataset	0.9424	0.9455	0.9426	0.9440	0.9773	0.9423	0.9426	0.9455	0.9393	0.0497	0.4884
External Dataset	0.8850	0.8595	0.9455	0.9004	0.9288	0.8111	0.9455	0.8595	0.9241	0.0997	0.5094

The model outperformed the traditional Framingham risk score (ΔAUC = + 0.1583, *P* < 0.001) (24) and surpassed reported deep learning models (AUC = 0.93) (25). With an AUC >0.9 indicating excellent performance, the near-perfect AUC of 0.9773 underscores its suitability for high-risk population screening. The steep ascent of the ROC curve in the upper-left quadrant reflects high true positive rates at low false positive rates, ideal for clinical applications.

**Figure 4:**
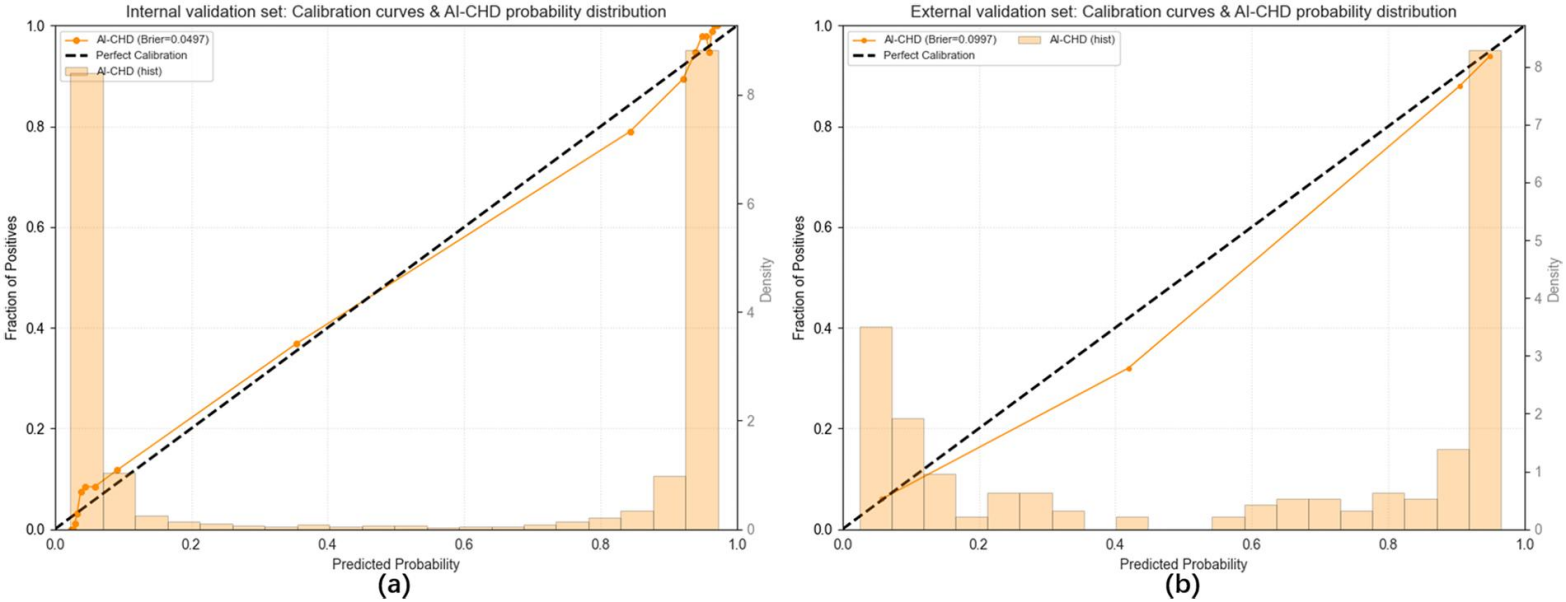
Calibration curves and predicted probability distributions of the AI-CHD model on the internal **(a)** and external **(b)** validation sets.

The calibration performance of the AI-CHD model is presented in Figures 4a,b, which illustrate the calibration curves and predicted probability distributions for the internal (a) and external (b) validation sets. In the internal validation set, the calibration curve closely aligns with the diagonal, with a Brier score of 0.0497, and the mean predicted probability closely matches the observed event rate. In the external validation set, the calibration curve remains well calibrated, and the mean predicted probability is consistent with the external event rate. These findings indicate that the AI-CHD model provides highly accurate and robust probability estimates across different populations.

**Figure 5:**
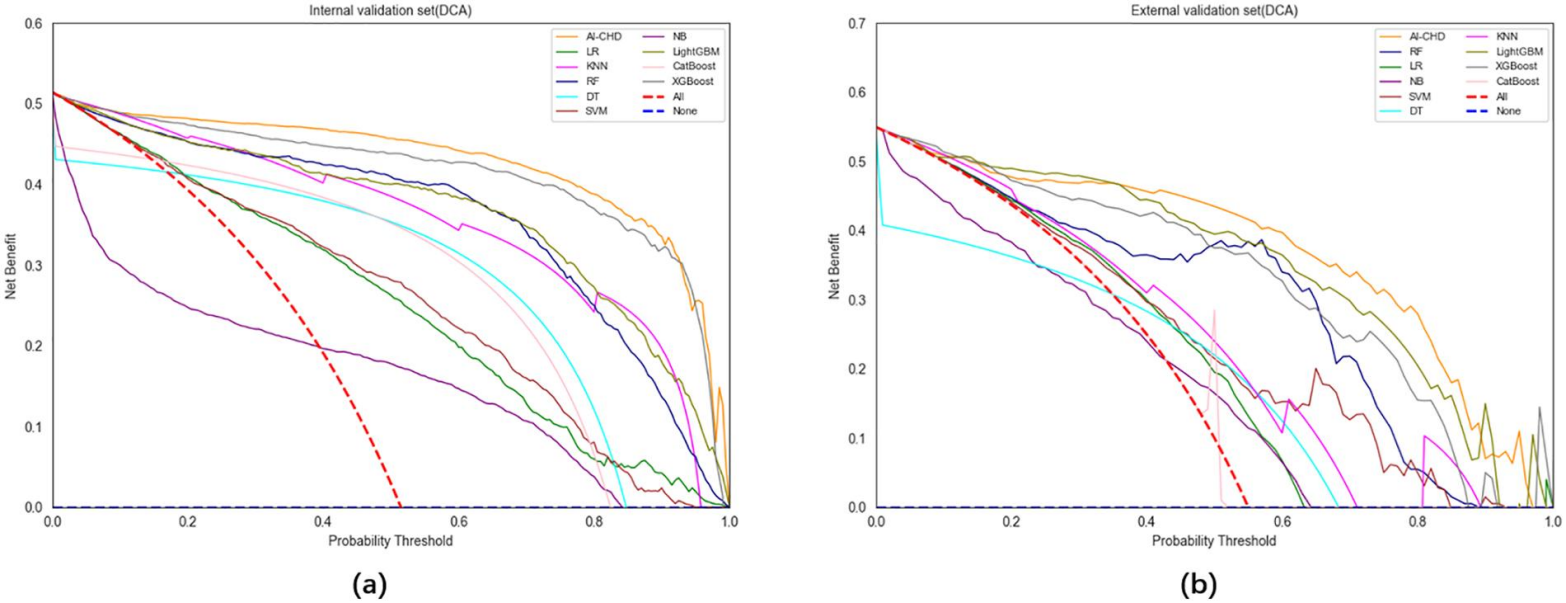
Decision curve analysis (DCA) of the model on the internal **(a)** and external **(b)** validation sets.

The decision curve analysis (DCA) illustrates the net benefit trends across threshold probabilities (Figures 5a,b). Among all models, the AI-CHD model achieved a net benefit of 0.4884 in the internal validation set and 0.5094 in the external validation set, demonstrating superior or comparable performance relative to other models. Together with its favorable AUC and low Brier scores, these results fully confirm the stable and reliable predictive performance and high clinical utility of the AI-CHD model, particularly for early risk screening and risk stratification of coronary heart disease in resource-limited settings.

### 3.4 Model interpretability analysis

#### 3.4.1 Global feature analysis

**Figure 6:**
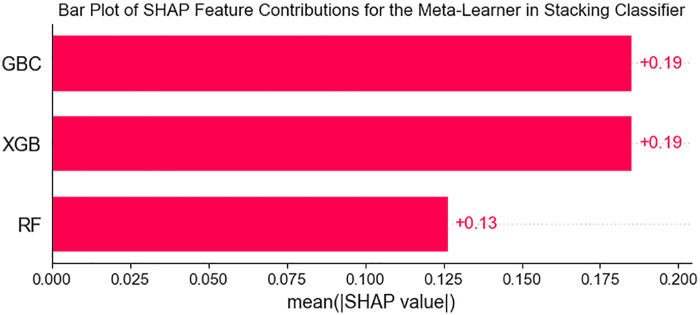
Marginal contributions of base models to ensemble decisions.

**Figure 7:**
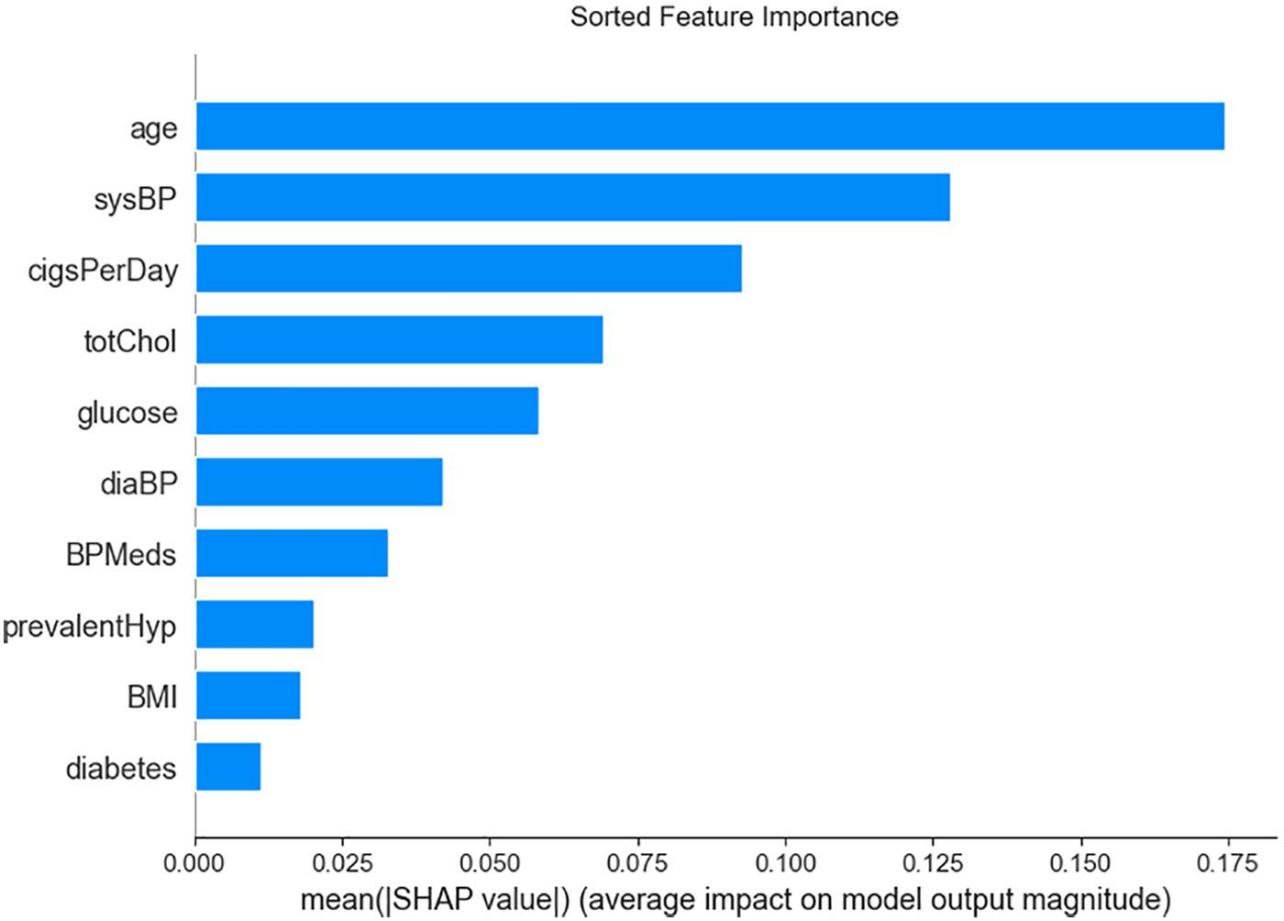
Feature importance rankings.

SHAP-based analysis of model decision mechanisms revealed that Gradient Boosting (GBC) and XGBoost exerted significant model-level contributions to the meta-learner (SHAP value: +0.19, 95% CI: 0.18–0.19), markedly outperforming Random Forest in terms of predictive contribution (SHAP value: +0.13) (Figure 6). At the feature importance level, age, smoking history, systolic blood pressure (sysBP), total cholesterol (totChol), and glucose were identified as key contributing features in the model, demonstrating substantial positive model-derived associations with CHD risk prediction (Figure 7).

**Figure 8:**
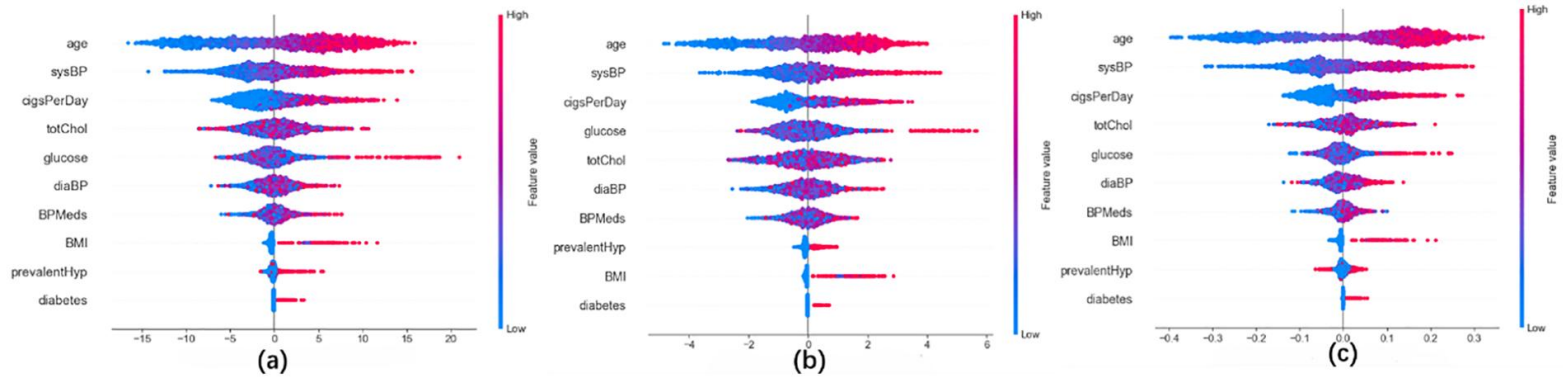
Feature-Response relationships across models gradient boosting **(a)**, XGBoost **(b)**, random forest **(c)**, illustrating positive/negative associations between features and predictions.

Heterogeneity analysis highlighted distinct feature dependencies: Gradient Boosting Trees (GBC) and Random Forest (RF) exhibited strong reliance on metabolic syndrome-related markers (totChol, BMI), whereas XGBoost showed specificity for glucose metabolism indicators. Notably, RF underperformed in associating hypertension history (prevalentHyp) with CHD risk (SHAP value = 0.062 vs. ensemble mean = 0.321) (Figures 8A–C).

#### 3.4.2 Predictors and synergistic effects

**Figure 9:**
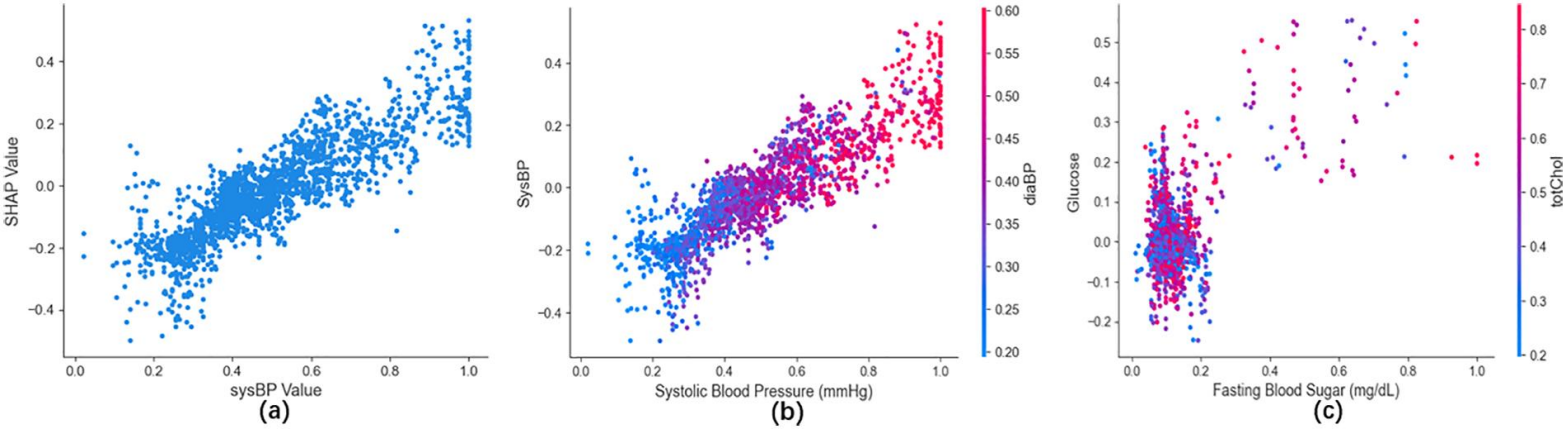
SHAP analysis of Key predictors. sysBP SHAP values **(a)**; sysBP-diaBP synergy **(b)**; glucose-totChol synergy **(c).**

A synergistic interaction pattern observed in the model between glucose and totChol amplified the model-predicted signal of metabolic dysregulation (Figure 9C), driving an exponential increase in predicted risk (*β* = 0.47). This model-derived pattern was highly consistent with ADA criteria for metabolic syndrome (26), suggesting that the model captured potential synergistic benefits of combined glucose-lipid management.

SHAP dependency analysis uncovered model-driven nonlinear dose-response association patterns. Systolic blood pressure (sysBP) emerged as the strongest contributing feature in model prediction, with SHAP values increasing markedly when sysBP exceeded 130 mmHg (ΔSHAP = 0.32 ± 0.07, 95% CI: 0.28–0.36, *P* < 0.001) (Figure 9A), and this model-derived threshold was highly consistent with the ACC/AHA threshold for Stage 1 hypertension (27). A positive model-derived association between sysBP and diastolic blood pressure (Figure 9B) supported the consistency between model outputs and pathophysiological knowledge.

### 3.5 Subgroup analysis

In order to clarify the clinical applicability of the model constructed in this study, comprehensive subgroup analysis was conducted based on the training set (*n* = 4,418) data, focusing on 9 core clinical features: age, blood pressure (systolic blood pressure, diastolic blood pressure), glucose, total cholesterol, cigarettes per day, BMI, prevalent hypertension, diabetes, and BP medication use. The CHD positive rate was used as the core evaluation index for all subgroups to analyze the predictive applicability of the model in different populations, and the specific results are shown in Table 5 (detailed data of subgroup analysis).

**Table 5:** Subgroup analysis of CHD positive rate in the training Set.

Subgroup category	Subgroup	Sample size	CHD positive	Positive rate (%)
Age Group	<45	1,359	239	17.6
	45–55	1,606	840	52.3
	55–65	1,320	995	75.4
	≥65	133	107	80.5
sysBP	<120	1,259	345	27.4
	120–139	1,708	777	45.5
	140–159	855	570	66.7
	≥160	596	489	82
diaBP	<80	1,725	654	37.9
	80–89	1,560	757	48.5
	90–99	737	463	62.8
	≥100	396	307	77.5
Glucose	<100	4,044	1,940	48
	100–125	243	122	50.2
	≥126	131	119	90.8
totChol	<200	874	287	32.8
	200–239	1,498	716	47.8
	≥240	2,046	1,178	57.6
Cigarettes Per Day	Light	2,777	1,300	46.8
	Moderate	484	272	56.2
	Heavy	1,157	609	52.6
BMI	Underweight	29	10	34.5
	Normal	1,934	804	41.6
	Overweight	1,994	1,087	54.5
	Obese	461	280	60.7
prevalentHyp	No	3,160	1,305	41.3
	Yes	1,258	876	69.6
Diabetes	No	4,317	2,098	48.6
	Yes	101	83	82.2
(BPMeds)	No	4,136	1,931	46.7
	Yes	47	31	66

As shown in Table 5, the CHD positive rate showed significant distribution differences among all subgroups, which was highly consistent with the clinical incidence rule of cardiovascular diseases: in the age subgroup, the CHD positive rate increased significantly with age, with only 17.6% in the <45 years group and as high as 80.5% in the ≥65 years group, indicating that the model has stronger predictive pertinence in the middle-aged and elderly populations; in the blood pressure-related subgroups, the CHD positive rates of the systolic blood pressure ≥160 group and diastolic blood pressure ≥100 group were 82.0% and 77.5%, respectively, which were significantly higher than those of the normal blood pressure group, suggesting that the model has good predictive efficacy for CHD risk in hypertensive populations; in the glucose subgroup, the CHD positive rate of the glucose ≥126 group was 90.8%, which was much higher than that of the normal glucose group (48.0%), indicating that the model can effectively identify the CHD incidence risk in hyperglycemic populations; in the BMI subgroup, the positive rate of the obese group (BMI≥30) was 66.0%, which was significantly higher than that of the normal weight group (41.6%), consistent with the clinical cognition that obesity increases the risk of cardiovascular diseases; in addition, the CHD positive rates of populations with hypertension and diabetes were 69.6% and 82.2%, respectively, which were significantly higher than those of non-diseased populations, and the positive rate of BP medication users (66.0%) was higher than that of non-users (46.7%).

In the subgroup of cigarettes per day, the CHD positive rate was the highest in the moderate smoking group (10–20 cigarettes per day, 56.2%) and the lowest in the light smoking group (0–10 cigarettes per day, 46.8%), but there was no obvious regularity in the overall difference, which may be related to the range of smoking quantity grouping and sample distribution. It should be noted that some subgroups (such as the BMI underweight group and BP medication use group) had small sample sizes (29 cases and 47 cases, respectively), and their results need to be interpreted cautiously in combination with clinical practice, but they do not affect the overall conclusion of the subgroup analysis.

In summary, the CHD prediction model constructed in this study provides descriptive evidence of CHD distribution across clinically relevant strata, especially in middle-aged and elderly populations (≥45 years old), hypertensive, diabetic, hyperglycemic, obese and other high cardiovascular risk populations, which should not be overinterpreted as subgroup-specific performance validation of the model and provides data support for subsequent clinical promotion and application.

### 3.6 Comparison of baseline characteristics between training and external validation sets

Statistical comparisons of baseline characteristics between the training set (*n* = 4,418) and the external validation set (*n* = 200) were performed to assess population differences, as shown in Table 6. Significant differences were observed in age, diaBP, glucose, totChol, cigsPerDay, BMI, and BPMeds (all *P* < 0.001). Patients in the external validation set were significantly older, with higher glucose levels, lower cholesterol levels, fewer smoking exposure, higher prevalence of hypertension and diabetes, and higher usage of antihypertensive medications. These differences reflect the real-world clinical scenario where patients seeking medical care tend to be older and carry more comorbidities.

**Table 6:** Baseline characteristics comparison between the training set and external validation set.

Variable	Training set (*n* = 4,418)	External validation Set (*n* = 200)	*P*-value
sysBP	133.51 ± 21.17	133.51 ± 18.95	0.996
age	49.99 ± 8.33	66.17 ± 10.50	0
diaBP	83.44 ± 12.09	75.77 ± 10.31	0
glucose	82.76 ± 26.41	106.04 ± 46.67	0
totChol	237.37 ± 42.57	165.87 ± 42.68	0
cigsPerDay	9.04 ± 11.64	1.24 ± 5.17	0
prevalentHyp	1,258 (28.5%)	115 (57.5%)	0
diabetes	101 (2.3%)	55 (27.5%)	0
BMI	25.84 ± 3.70	24.07 ± 3.07	0
BPMeds	0.04 ± 0.16	0.57 ± 0.50	0

Although notable baseline differences existed between the two cohorts, all differences were explicitly identified and statistically tested rather than ignored or artificially adjusted. Preserving the original distribution of the external cohort ensures the authenticity and independence of external validation, which is critical for evaluating the generalization ability of the prediction model.

### 3.7 Clinical validation

This study has been clinically validation (Nantong University Affiliated Hospital cohort, *n* = 200) confirmed the model's robust generalizability: Accuracy = 0.8850 (95% CI: 0.8424–0.9226), AUC = 0.9288 (95% CI: 0.8852–0.9607), Sensitivity/Specificity: 0.9455 (95% CI: 0.9107–0.9856)/0.8111 (95% CI: 0.7845–0.8705).

A total of 200 consecutive patients were enrolled, with a CHD prevalence of 50%, ensuring balanced predictive performance for model evaluation. The cohort had a mean age of 65.8 ± 10.2 years and mean systolic blood pressure of 137.5 ± 18.3 mmHg. The distributions of hypertension (54.0%), diabetes (29.5%), and dyslipidemia were consistent with real-world characteristics. Although minor baseline differences existed between cohorts, these reflected genuine clinical heterogeneity rather than selection bias. The model achieved stable and reliable performance, supporting its generalizability. Given the cohort's representative composition and balanced event rate, 200 cases suffice to provide credible real-world evidence for model performance.

Significant clinical net benefit was demonstrated via net reclassification improvement (NRI = 0.5990, 95% CI: 0.4723–0.7204, *P* < 0.01) and integrated discrimination improvement (IDI = 0.3505, 95% CI: 0.2962–0.4075, *P* < 0.01). The stacking architecture effectively minimized false-positive overtesting and false-negative underdiagnosis, ensuring timely interventions for high-risk patients.

## 4 Discussion

This study integrates machine learning with SHAP explainability to develop AI-CHD, a stacked ensemble model (GBC, RF, XGBoost), and an interactive clinical platform. Internal validation (AUC = 0.9773, accuracy = 0.9424) and external validation (accuracy = 0.8862) demonstrated superior performance. SHAP analysis elucidated nonlinear effects and synergistic mechanisms of sysBP, age, and glucose, aligning with ACC/AHA guidelines. The platform's reliance on 10 routinely accessible indicators (sensitivity = 0.9395) enables a paradigm shift from population-based statistics to individualized precision medicine.

Advantages Over Existing Studies:In terms of performance, AI-CHD outperforms the previously reported deep learning models. A systematic review and meta-analysis (25) showed that the pooled AUC of boosting algorithms (e.g., GBC, XGBoost) for coronary heart disease prediction was 0.88 (95% CI 0.84–0.91), while the pooled AUC of custom algorithms was 0.93 (95% CI 0.85–0.97). AI-CHD achieved an AUC of 0.9773 (95% CI: 0.9563–0.9789) in internal validation, which exceeds the pooled performance reported above and demonstrates superior discriminative ability. In terms of transparency, existing artificial neural network (ANN) models can achieve high prediction accuracy (96.25%) (16), but their clinical interpretability is limited due to the “black-box” nature. In contrast, AI-CHD quantifies the contribution of each feature to the prediction results via SHAP, which effectively addresses the limitations of black-box models and significantly enhances the clinical interpretability of the model and physician confidence. In terms of generalizability, cross-center validation confirmed the model's adaptability across diverse populations. Regarding clinical utility, in contrast to existing prediction models that rely on specialized measurements such as carotid ultrasound parameters (e.g., carotid intima thickness and stiffness coefficient) (6), or extensive laboratory panels including 50 routine blood and biochemical features (7), AI-CHD requires only 10 routine clinical indicators (e.g., blood pressure, glucose, and lipids) without additional imaging or specialized equipment. This significantly reduces barriers to implementation. Although the aforementioned model achieved excellent performance (XGBoost AUC = 0.9921), AI-CHD achieves high predictive efficiency with a far simpler input set, making it more suitable for primary care settings and resource-limited environments while maintaining superior accuracy.

This study has several limitations that should be explicitly acknowledged to ensure objectivity and rigor, as well as to provide directions for future improvement. First, although AI-CHD achieved promising predictive performance, it did not explore deep learning architectures such as Convolutional Neural Networks (CNN) and Recurrent Neural Networks (RNN). These architectures possess unique advantages in capturing complex spatiotemporal patterns and are particularly suitable for longitudinal follow-up data and imaging-based multimodal data. Their absence not only restricts model applicability in settings where such data are available but may also prevent the model from fully exploiting potential complex relationships within the data, limiting further improvements in predictive performance. Second, the model was built solely on routine clinical parameters and did not incorporate mechanistic biomarkers, including inflammatory markers (e.g., hs-CRP, IL-6, TNF-α), oxidative stress markers (e.g., ox-LDL, MPO), and endothelial function markers (e.g., VCAM-1, ICAM-1). This implies that the model cannot fully capture the depth and complexity of current understanding of coronary heart disease pathogenesis, remaining limited to phenotypic prediction rather than mechanism-linked inference. It may underestimate risk in certain subgroups with occult coronary heart disease and restricts the model's ability to interpret disease mechanisms. Third, the model relies on static clinical parameters measured at a single time point and lacks integration of dynamically monitored data, such as continuous glucose levels, ambulatory blood pressure trends, and long-term lipid fluctuations. This limits the ability to capture temporal features of disease progression and may reduce predictive accuracy when patients' clinical status fluctuates, hindering dynamic early warning of disease evolution. Fourth, external validation was performed using only one independent cohort; future studies will require more extensive multi-center validation across diverse geographic regions and healthcare settings to further establish the model's generalizability.

Furthermore, to contextualize this work within cardiovascular pathophysiology, we acknowledge that our predictive modeling focused exclusively on routine clinical phenotypic indicators and did not incorporate the systematic mechanistic framework of coronary heart disease pathogenesis. The system-level Petri-net framework for atherosclerosis proposed by Formanowicz et al. reveals core regulatory mechanisms in lesion development (28); studies on inflammasome regulation and NF-*κ*B-driven innate immune activation further highlight the complex biological network underlying CHD progression, which serves as a critical link between biomarkers and clinical phenotypes. Although such mechanistic aspects lie beyond the scope of this predictive modeling study, their explicit recognition helps interpret the model's limitations and provides a roadmap for future integration of mechanistic knowledge with machine learning. This will enable the construction of an integrated phenotype–mechanism–prediction model to enhance biological plausibility and clinical translational value.

Therefore, the primary objective of AI-CHD is to support rapid risk stratification of coronary heart disease (CHD) by non-specialist clinicians in resource-limited settings and to assist in urgent therapeutic decision-making. These innovations demonstrate that this study, through its comprehensive “mechanism analysis-model optimization-clinical translation” pipeline, provides a tool for CHD prevention and management that balances high accuracy with interpretability. Future integration of multimodal data (e.g., genomics, wearable device metrics) and dynamic prediction modules will further advance the transformation of cardiovascular disease management from a reactive “treatment-oriented” paradigm to a proactive “prevention-first” framework. Given that AI-CHD relies on simple, routinely accessible clinical parameters, we anticipate its pivotal role in CHD risk assessment. By enabling preliminary diagnosis during the laboratory confirmation process, it offers timely guidance for initial interventions.

From a methodological perspective, while the data-driven machine learning framework employed in this study achieved favorable predictive performance, we recognize the existence of complementary systems-level modeling approaches that could offer alternative perspectives for cardiovascular risk modeling (29). For instance, Viability Theory provides a formal framework for analyzing constrained physiological trajectories, which can help understand the boundary conditions governing transitions between pathological states (30). Additionally, Petri nets serve as a mathematical structure capable of representing concurrency, inhibition, and feedback mechanisms in biological networks, and have been successfully applied to model inflammatory signaling pathways and atherosclerotic regulatory networks (31). Although these approaches fall outside the scope of the present study, acknowledging their existence helps broaden the conceptual framing and points toward future directions for integrating mechanistic knowledge with machine learning to develop more biologically plausible predictive models.

## 5 Conclusion

The AI-CHD risk prediction model developed in this study leverages interpretable machine learning techniques to effectively identify high-risk populations exceeding clinical thresholds in key biomarkers, including blood pressure, glucose, and lipid levels. The interactive prediction platform enables individualized risk profiling, providing transparent decision support for clinicians and optimizing healthcare resource allocation. By enhancing patient self-management awareness and offering an evidence-based foundation for public health policy formulation, this system pioneers a paradigm shift in CHD prevention—from population-based paradigms to individualized precision medicine.

## Data Availability

The datasets presented in this study can be found in online repositories. The names of the repository/repositories and accession number(s) can be found below: The source code of the study design is available at https://github.com/Huan-star/CHD_Prediction.
